# The Role of miRNA Expression in Congenital Heart Disease: Insights into the Mechanisms and Biomarker Potential

**DOI:** 10.3390/children12050611

**Published:** 2025-05-07

**Authors:** Savina Mannarino, Valeria Calcaterra, Filippo Puricelli, Giulia Cecconi, Claudia Chillemi, Irene Raso, Erika Cordaro, Gianvincenzo Zuccotti

**Affiliations:** 1 Pediatric Cardiology Unit, Pediatric Department, Buzzi Children’s Hospital, 20154 Milan, Italy; savina.mannarino@asst-fbf-sacco.it (S.M.); filippo.puricelli@asst-fbf-sacco.it (F.P.); giulia.cecconi@asst-fbf-sacco.it (G.C.); claudia.chillemi@unimi.it (C.C.); irene-raso@asst-fbf-sacco.it (I.R.); 2 Department of Internal Medicine and Therapeutics, University of Pavia, 27100 Pavia, Italy; erika.cordaro01@universitadipavia.it; 3 Pediatric Unit, Pediatric Department, Buzzi Children’s Hospital, 20154 Milano, Italy; gianvincenzo.zuccotti@unimi.it; 4 Department of Biomedical and Clinical Science, University of Milano, 20157 Milano, Italy

**Keywords:** epigenetics, miRNA, congenital heart disease, cardiac malformation, fetal heart development

## Abstract

Congenital heart diseases (CHDs) are among the most common congenital malformations. Despite significant advancements in understanding the embryonic development of the heart, the etiology of CHDs remains largely unknown. The complexity of the processes involved in heart formation limits our ability to identify all molecular mechanisms underlying CHDs. Recently, microRNAs (miRNAs) have provided new insights into the molecular mechanisms of CHDs. This narrative review evaluates the evidence linking expression to CHDs and discusses the potential of RNA expression regulation as a promising avenue for therapeutic biomarker development. A search of the literature, focusing on the role of miRNAs in CHDs, was carried out to identify pertinent studies published over the last decade. The literature search was performed utilizing the PubMed and Scopus databases. The selection criteria included peer-reviewed original studies, clinical research, meta-analyses, and review articles written in English. Multiple investigations have highlighted the essential role of miRNAs in cardiac development and function, showing that their distinct expression patterns can broadly and specifically influence cellular signaling pathways involved in heart abnormalities. The regulation of mRNA expression emerges as a key factor in the pathogenesis of CHD, paving the way for the identification of novel molecular biomarkers. Alterations in transcriptional profiles could offer innovative and highly specific tools for risk stratification and the clinical monitoring of patients. In conclusion, although further studies are needed to validate the efficacy and clinical applicability of these biomarkers, the mRNA-based approach stands out as a promising perspective for precision medicine in the CHD context.

## 1. Introduction

Congenital heart diseases (CHDs) are one of the most common types of congenital malformations, with an estimated incidence of approximately 8 in 1000 live births [1]. They include a wide range of malformations that originate early during fetal heart development such as cardiac septation defects, conotruncal and aortic arch anomalies, right and left outflow obstructive defects, and lateralization defects.

Despite significant advancements in our understanding of the embryonic development of the heart, the etiology of CHDs remains largely unknown. The complexity of the processes involved in heart formation limits our ability to easily identify the molecular mechanisms underlying CHDs. Heart development is tightly regulated by the precise temporal and spatial expression of various genes within a hierarchical regulatory framework [2]. Analyses through whole-genome and exome sequencing have enabled the discovery of nearly 400 genes, including those encoding transcription factors and regulatory elements critical for heart development. To date, around 35% of CHDs can be attributed to genetic origins [3]. Specifically, this includes 3–5% of monogenic disorders, 8–10% of chromosomal anomalies/aneuploidies, and 3–10% of pathogenic copy number variants (CNVs) in non-syndromic cases (up to 25% in syndromic cases) [1,2,3]. Environmental causes are identifiable in 2–3% of CHDs. However, the remaining 60% of CHDs have no identified cause, suggesting a multifactorial origin where extragenomic factors that modify gene expression may play a significant role [4].

Epigenetics, which refers to the extragenomic mechanisms that regulate gene expression without altering DNA sequences, is emerging as a potential factor in the etiology of heart disease [4,5]. Canonical epigenetic mechanisms include spatial regulation and the higher order chromatin structure, DNA methylation, histone modification, and the activity of certain non-coding RNA species [5]. These epigenetic changes can be transmitted across generations without involving permanent changes to the DNA sequence [4,5]. Unlike DNA sequence mutations, epigenetic modifications are reversible, allowing the epigenome to adapt in response to both intrauterine and extrauterine environmental factors throughout development.

Recent investigations into non-coding RNAs (ncRNAs), including long non-coding RNAs (lncRNAs) and microRNAs (miRNAs), have shed light on the molecular pathways underlying both congenital and acquired diseases [4]. Their role in gene expression regulation has been investigated in the context of congenital heart disease [4]. MiRNAs, in particular, have been specifically studied as potential diagnostic and prognostic molecular markers due to their stability in blood, urine, and other biological fluids, and their resistance to RNA-degrading enzymes [2,6].

This narrative review evaluates the evidence linking miRNA expression to CHDs and discusses the potential of RNA expression regulation as a promising avenue for therapeutic biomarker development.

## 2. Methods

A search of the literature, focusing on the role of miRNAs in CHDs, was carried out to identify pertinent studies published over the last decade (2015–2025). The search was conducted using the PubMed and Scopus databases. Keywords, which were applied individually or in combination, included congenital heart disease OR cardiac malformation OR congenital heart defects OR congenital cardiac malformations OR conotruncal defects OR ventricular septal defect OR atrial septal defects OR septation defects OR atrioventricular septal defect OR obstructive heart disease AND/OR Epigenetics OR miRNA OR microRNA OR miR AND/OR risk factors AND/OR heart development.

The inclusion criteria comprised original research articles, clinical trials, meta-analyses, and review papers. The exclusion criteria included letters, case reports, brief communications, and commentaries. Studies were limited to those published in English.

**Figure 1:**
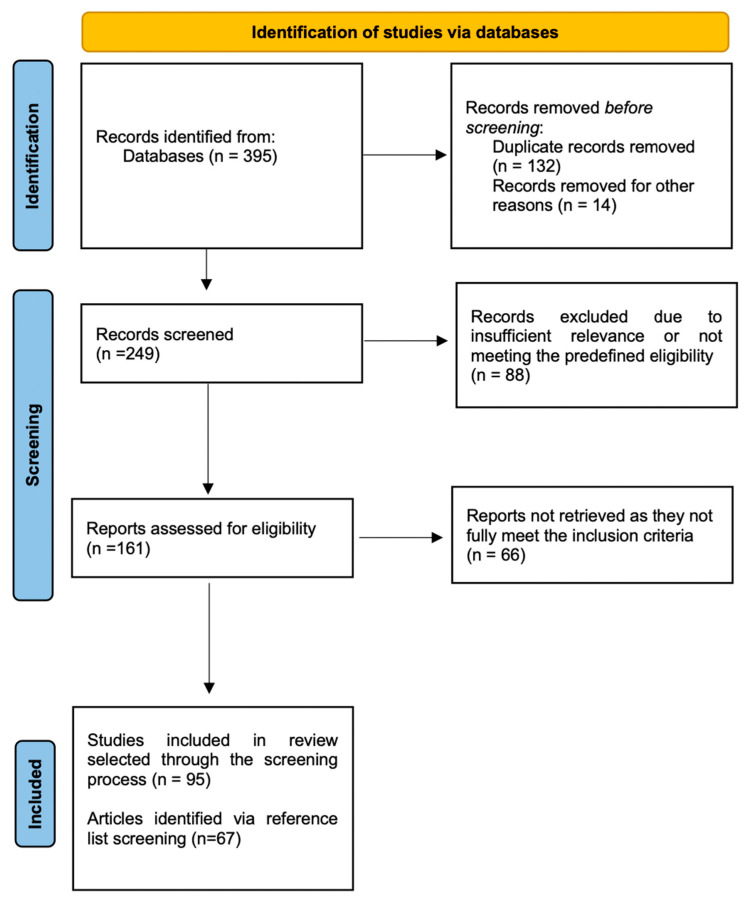
Flowchart of the selection process.

From the initial search, 395 records were retrieved. During the screening process, 146 records were excluded due to duplication, irrelevance (e.g., not original research, non-English language, not focused on humans, etc.). The remaining 249 titles and abstracts were evaluated, from which 161 full-text articles were reviewed. Of these, 95 were identified as relevant and were included for detailed analysis and discussion. A visual summary of the selection process is presented in Figure 1. Additionally, the reference lists of all articles were examined to identify relevant studies that could be included in the manuscript (n = 67), along with the references used for the introduction, general descriptive sections, and discussion (even if they were published more than 10 years ago and/or involving animal models).

## 3. Heart Development and Congenital Heart Diseases

### 3.1. Heart Development

Understanding the process of cardiogenesis is extremely important to unveil the pathogenesis of CHDs. The morphogenesis of the human heart begins during early cardiogenesis and continues through the organogenesis phase, with major structures forming between day 15 and 45 days after conception. Most major cardiac structures have been established in their spatial conformation by 30 days. Heart development is commonly divided into four key stages, each overlapping with the next [7]:

(1) Early cardiogenesis (days 8–18 of embryonic development): This developmental phase starts with the establishment of the cardiac fields and the crescent-like cardiac primordium during gastrulation, and concludes with the emergence of two endocardial tubes encased by myocardial cells.
(2) Morphogenetic phase (weeks 4–8 of embryonic development): This starts with the formation of the linear heart tube originating from the first heart field (FHF) and concludes with the assembly of all primordial components into the fully developed four-chambered heart, primarily derived from the second heart field (SHF).
(3) Septation and remodeling of the heart chamber (~day 30 of embryonic development): During this phase, differential growth and remodeling occur, forming the valves and septa, thereby defining the distinct identities of the atrial and ventricular cavities.
(4) Maturation and histodifferentiation (weeks 16–18 of embryonic development): this phase includes the maturation of the ventricular and atrial myocardium and the development of ventricular–arterial and atrio-ventricular valve systems, as well as the evolution of the conduction system and coronary vessels [7,8].

Cardiac development is a highly coordinated process that relies on the involvement of various cell lineages originating from distinct heart fields, as well as contributions from cardiac neural crest cells and cells of the pre-epicardial organ [7,8]. Cardiogenic precursors can be identified soon after gastrulation as two bilateral mesodermal cell subpopulations. At this early stage, they form two cardiogenic fields and then a crescent-shaped structure known as “cardiac crescent” from which the heart tube will be formed. The cardiac crescent is made up of two different cell populations, cardiocytes and endocardial endothelial cells, which are considered to be the first heart field [9]. Other cells form the secondary heart field and move towards the dorsal and cephalic parts of the heart tube [7,9]

The spatial arrangement of cells in the developing heart tube is closely associated with their eventual roles; early on, cell identity and cardiac asymmetry are established along a two-dimensional left–right axis within the crescent-shaped cardiac fields. These are refined during the tube formation phase, which involves more complex three-dimensional spatial regulation [7,8,9].

Following heart tube formation, the proper development of the heart depends on the efficient communication between the various cellular progenitors, including the FHF, SHF, and neural crest cells, throughout the subsequent stages of cardiogenesis [7,8,9]. Disruptions in cellular migration, interactions, apoptosis regulation, or proliferation can lead to misaligned structures, impaired tissue growth, and altered blood flow, contributing to the wide range of congenital heart defects (CHDs) [5].

During embryonic development, the lateral folding of the embryo brings the two bilateral heart fields together at the midline, where they fuse to form a single primitive heart tube. This tube can be anatomically divided into three main regions: the atrium, the ventricle, and the bulbus cordis [10]. The cardiac tube subsequently displays a looping process. The primary heart tube undergoes a clockwise rotation which is the first sign of embryonic asymmetry. It is then possible to distinguish the bulbus cordis and the ventriculobulbar folds by the bending originating in the ventriculobulbar groove. Afterwards, the ventricular parts undergo a septation process which forms the two ventricles [10].

The looping phase of heart development appears complex and multi-staged [7,8,9,10]. It is of crucial importance since it determines the position of the ventricles in relation to the atria and the alteration of this process leads to the formation of many severe congenital heart diseases [7,8,9,10]. When looping is concluded, the primary tube appears as a formed heart, in which it is possible to distinguish a cephalic segment, the arterial trunk with the atria, and a caudal portion made by the ventricle [7,8,9,10].

The atrial and ventricular chambers undergo gradual maturation, characterized by the development of trabecular and compact layers in the ventricles and the formation of pectinate muscles in the atria [10].

Endocardial cushions are subsequently formed at the atrioventricular and conotruncal regions from which the primitive cardiac valves originate. This process, which involves neural crest cells, begins with the thickening of the margins surrounding the atrioventricular openings [7,8,9,10].

The embryonic heart chambers and major vessels undergo a complex septation process, resulting in a fully formed four-chambered heart with distinct inflow and outflow tracts. Concurrently, the heart tube is partitioned at the level of the arterial trunk and bulbus cordis, separating the ascending aorta from the pulmonary trunk. This arterial trunk subsequently bifurcates into two distinct vessels: an anterior vessel connecting to the right ventricle, and a posterior vessel opening into the left ventricle. Furthermore, a specialized cardiac conduction system with slow conducting nodes, the sinoatrial and the atrioventricular nodes, respectively, and fast conducting branches, such as the common branch of the bundle of His and the left and right bundle branches, is also configured [11].

The culmination of these tightly regulated embryonic processes results in the proper formation of the heart. Conversely, disruptions, whether genetic, epigenetic, or environmental in origin, can lead to congenital heart defects.

### 3.2. Pathogenesis of Congenital Heart Diseases

CHDs can be defined as structural heart defects present before and/or at birth. With a prevalence of 8 in every 1000 infants they represent the most frequent of all congenital malformations and account for over 40% of prenatal deaths [12].

CHDs are also a leading cause of infant morbidity and mortality with an incidence of 1–2% in newborn patients.

CHDs include different phenotypes and can be broadly classified in the following:

- Right-sided obstructions: pulmonary stenosis (PA), tricuspid atresia (TA), and pulmonary atresia (PA);
- Left-sided obstruction defects: including hypoplastic left heart syndrome (HLHS), mitral stenosis, aortic stenosis, aortic coarctation, and interrupted aortic arch;
- Septation defects: ineffective separation of the atria (atrial septal defects, ASDs), the ventricles (ventricular septal defects, VSDs), or both (atrioventricular septal defects, AVSDs);
- Conotruncal defects: transposition of the great arteries (TGA), double outlet right ventricle (DORV), Tetralogy of Fallot (TOF), and persistent truncus arteriosus (PTA).

As already mentioned, heart formation takes place in the early phases of the prenatal period. Both genetic, environmental, and epigenetic factors contribute to cardiac malformation.

#### 3.2.1. Genetic Factors

Studies report different data regarding causative genetic alteration in CHDs, varying between <20% of cases and 35–40% [13,14].

Genetic screening of CHDs, particularly in familial cases, has provided valuable insights into their hereditary basis. The occurrence of CHDs among first-degree relatives is notably higher (22%) compared to second-degree relatives (3.4%). Furthermore, concordance rates observed in familial clusters revealed a 94.4% match among identical twins and 33.3% among dizygotic twins [15].

CHDs can also be present in syndromic patterns with genetic associations [13,14,15]. The most studied and well-known genetic defects are autosomal single and multi-gene mutations that cause the loss or gain of function of a certain protein [2,13,14,15]. Moreover, CHDs are associated with chromosomal anomalies such as trisomies 13, 15, 18, and 21 and 22q11.2 deletion [13,14]

Notwithstanding that the pathogenetic mechanisms of CHDs remain largely unclear, several transcriptional factors (TFs) have been recognized as being part of cardiogenesis. This is a complex process controlled by several mechanisms that coordinate different cells groups.

As reported by Lien et al. [16], another important TF in the early stage is the growth factor CR-1, which stimulates the activity of the atrial natriuretic factor [17]. The specialization of a cardiac progenitor is mediated by the expression of NKX2-5 and its abnormalities cause defects of heart formation.

The process of cardiac differentiation is regulated by signaling molecules such as fibroblast growth factors (FGFs), bone morphogenetic proteins (BMPs), Wnt family proteins, and key transcription factors including TBX5, GATA4, and BAF60C. Among these, GATA4 has been particularly well characterized for its essential function in proper heart development [18]. In fact, GATA4 is widely expressed in cardiomyocytes where it regulates gene expression by interacting with other TFs: NKX2-5, MEF2A, MEF2C, SRF, TBX5, and TEAD1 [19].

Cells of the first and second heart fields appear to be pre-determined to produce left- and right-sided structures and the role of PITX2 has been demonstrated in this left/right patterning [20].

The fusion of the heart tube is associated with the expression of the VHMC-1 gene of cardiac myosin heavy chains [21], while genes encoding for light and heavy myosin chains (MLC-2V, MLC-2A, and A-MHC) present themselves at a later time in ventricular and atrial cardiomyocytes. Heart tube bending and twisting are then probably regulated by the unbalanced distribution of cells which highly express the XIN gene [22]. At a later stage of maturation, different T-box transcriptional factors contribute to regulating cardiac chamber modeling, including TBX-2, TBX-3, TBX5, and TBX20 [23]. In particular, TBX5 and TBX20 cooperate with NKX2-5 and GATA4 to drive myocardial differentiation, contributing to the development of the atrioventricular openings as well as the formation of the mitral and tricuspid valves [24].

Other factors, like vascular endothelial growth factor (VEGF), fibroblast growth factor (FGF), and platelet-derived growth factor, regulate vascular processes and coronary artery formation together with substances such as erythropoietin and retinoic acid [25].

#### 3.2.2. Environmental Factors

Environmental factors play a crucial role in the development of CHDs, as they can influence fetal development during critical periods of organ formation. These factors may include maternal conditions, exposure to toxins or certain medications, nutritional deficiencies, and the lifestyle habits of parents [6]. A population-based study conducted across 24 European countries found a significant link between maternal age and the overall incidence of congenital heart disease (CHD), with increased prevalence observed among both younger mothers (≤24 years) and older mothers (35–44 years) [26]. The findings indicated that younger maternal age was more frequently associated with severe CHD forms, whereas advanced maternal age tended to correlate with milder presentations [26]. Additionally, the specific stage of gestation at which developmental disruptions occur plays a crucial role in determining both the severity and extent of CHD-related impairments [27].

The elevated risk of CHDs associated with advanced maternal age has been linked to several biological factors, including oocyte aging, diminished sperm quality, compromised genetic integrity, and a decline in cellular repair mechanisms [28].

Maternal medical condition also represents a risk factor. It was reported that pregestational diabetes in women induced a three-fold increased risk of developing CHDs in the fetus, while the risk percentage reached 47% in women with gestational diabetes [29]. A nationwide cohort study demonstrated that maternal diabetes induced fetal hyperglycemia, inducing a teratogenic effect that increased the risk of CHDs in the infant [30]. It was observed that there was a positive association between maternal obesity and both defects of the great vessels [31] and an increased risk of CHDs [32] in the fetus. The link between gestational diabetes and CHDs is not yet fully elucidated. Dong et al. [33] showed that in diabetic mice, a total of 149 mapped miRNAs in the developing heart were significantly altered by pregestational diabetes mellitus (PGDM). A majority of predicted miRNA target genes appeared to be linked with pathways essential for cardiac development, such as STAT3 and IGF-1 signaling, as well as transcription factors including Cited2, Zeb2, Mef2c, Smad4, and Ets1. Notably, the overexpression of the antioxidant enzyme superoxide dismutase 1 was found to counteract the miRNA alterations induced by PGDM, implying a significant role of oxidative stress in miRNA dysregulation [33].

Basu et al. [34] showed the haploinsufficiency of Notch1, a key transcriptional regulator known to cause CHDs. Maternal hyperglycemia appeared to impair nitric oxide (NO) production by limiting chromatin accessibility at the endothelial NO synthase (Nos3) gene locus and enhancing the expression of Jarid2, a component of histone methyltransferase regulatory complexes. This elevation in Jarid2 levels subsequently suppressed Notch1 gene expression.

Additionally, maternal comorbidities associated with CHD, including pregestational diabetes and preeclampsia, are linked to abnormal placental development, structure, and function, suggesting a connection between the placenta and the heart. The so-called ‘Placenta–Heart axis’ refers to the parallel development of the placenta and heart—critical vascular structures that share a common ontogeny [35]. Early impairments in placental shape, size, cord insertion, and overall efficiency may influence cardiac development, and vice versa. The overlap between the embryonic heart and the developing placenta raises the possibility that a common etiological insult may lead to multiorgan dysfunction [35,36]. In this context, O’Hare et al. [36] showed that placental delayed villous maturation and maternal vascular malperfusion were more common in pregnancies with fetal CHDs than demographically matched controls.

Furthermore, a placental main nutrient transport drop induces oxidative stress, leading to cellular senescence and vascular dysfunction within the organ. While a certain degree of hypoxia is essential for angiogenesis in the fetus and placenta, an imbalance between oxidative stress and angiogenic signaling can disrupt normal fetal organ development [37].

Gestational hypoxia exerts significant negative effects on heart development, contributing to a higher incidence of congenital heart defects (CHDs). Recent research has shed light on the mechanisms through which hypoxic stress influences the fate of cardiac progenitor cells (CPCs). Hypoxia has been shown to modulate CPC proliferation and differentiation while inhibiting the maturation of cardiomyocytes [33]. Notably, hypoxic conditions lead to an upregulation of miR-210 in Sca-1^+^ CPCs, impeding their differentiation. Suppression of miR-210 expression, however, enhances the differentiation of these progenitor cells into cardiomyocytes. These results highlight the novel role of miR-210 in mediating hypoxia-related CHDs by altering CPC fates [38].

Additional conditions, such as maternal phenylketonuria, virus infections, and hyperthermia; and drug treatments, such as antidepressants [39] or specific hypertension medications [40], have also been reported to be correlated with a higher risk of developing CHDs, probably due to their teratogenic action [39,41,42,43].

Maternal malnutrition can also influence the development of CHDs. Deficiencies in folic acid during pregnancy can interfere with the formation of the neural tube and heart, leading to defects such as the transposition of the great arteries (TGA), Tetralogy of Fallot, and common arterial trunk [6,44]. Supplementing folic acid, particularly around conception, could reduce the risk of these defects [45]. Conversely, studies also reported no association between maternal periconceptional (specifically, from 4 weeks before and 8 weeks after conception) supplementation containing FA and CHD risk in infants [46,47].

Other environmental factors, such as pollutants; teratogenic drugs like thalidomide, lithium, and valproic acid; maternal smoking; and alcohol use, further increase the risk of CHDs by disrupting the gene expression and signaling pathways essential for heart development [6]. Specifically, smoking and alcohol can alter cardiogenic gene pathways, leading to defects like ventricular septal defects and atrial septal defects [48]. As reported by Kalisch-Smith JI, in alcohol consumption an abnormal methylation process is involved. a reduced expression of the histone methyltransferase G9α induces a reduction of histone H3K9me3 and a down regulation of the expression of some cardiomyogenesis-related genes (Mef2c, Cx43, Anp, and β-MCH) [43]. Interestingly, a protective effect of curcumin (a natural histone acetylation inhibitor) on alcohol-induced cardiac damage during pregnancy was demonstrated [49,50].

Chemical agents, such as retinoic acid, nitrophene, chlorinated hydrocarbons, and pesticides are potent teratogens that can also disrupt the heart development [51], as can dietary arsenic exposure [52]. Retinoic acid affects the secondary heart field and can lead to defects like double outlet right ventricle [53], dextroposition of the aorta, and TGA. Both excessive and insufficient levels of retinoic acid can impair heart formation and elevate the risk of CHDs [6].

Certain medications, including cyclooxygenase inhibitors [54], antiepileptics [55], and hormonal drugs [56], can also lead to CHDs. Ionizing radiation is another physical factor linked to heart defects [57].

In vitro fertilization (IVF), particularly the intracytoplasmic sperm injection (ICSI) technique, has been associated with an increased risk of CHDs [5,58]. The incidence of CHDs in children conceived through IVF/ICSI is 1.3%, compared to 0.68% in naturally conceived children [59]. Studies show the prevalence of CHDs to be 7.1% in IVF children, 9.9% in ICSI children, and 5.7% in naturally conceived children [60].

A recent study conducted by the Committee of Nordic ART Safety (CoNARTaS), involving a large cohort of 171,735 children born through assisted reproductive technology (ART), found an increased risk of major congenital heart defects (CHDs) compared to those conceived naturally. CHDs were diagnosed in 1.84% of children born after ART, whereas only 1.15% of spontaneously conceived children received a similar diagnosis, resulting in an adjusted odds ratio of 1.36. The study also highlighted that multiple pregnancies, regardless of the mode of conception, further elevated the risk [61].

Furthermore, paternal conditions should also be considered. Conflicting assessments have been reported regarding the influence exerted by paternal age, as studies have identified both advanced and younger paternal age as contributing factors for CHDs [44], but analyses detecting no association between advancing paternal age and CHDs have also been reported. Diabetes, hypertension, and viral infection as part of the paternal medical condition are potential risk factors for CHDs in offspring [62]. Furthermore, a dose-dependent correlation between paternal smoking and infants with CHDs [62] was found. A meta-analysis highlighted how strong paternal exposure to chemical agents or drugs increased the risk of CHDs [62]. It was reported that paternal consumption of marijuana led to an increased risk of the development of VSDs among their infants [39]. Furthermore, an increased incidence of CHDs was detected in children whose fathers worked as factory workers, janitors, or painters [62], probably due to the exposure to CHD-risk-associated chemicals such as phthalates and polychlorinated or alkylphenolic compounds [63].

#### 3.2.3. Gene Expression and Epigenetics

**Figure 2:**
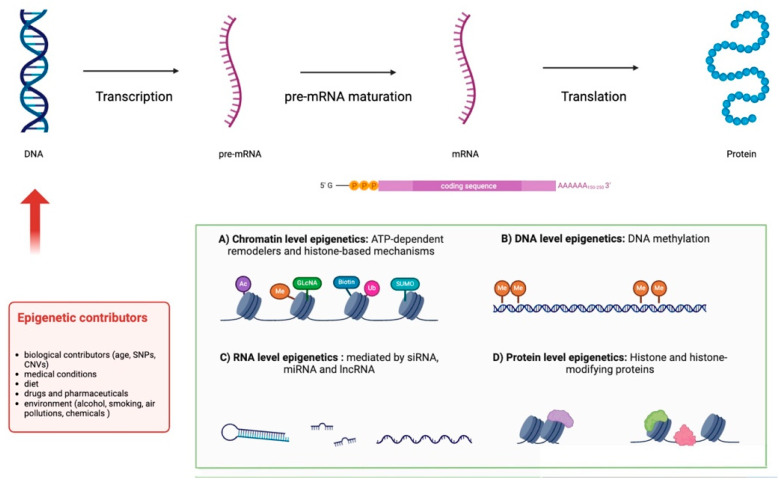
Transcription of DNA into mRNA and ncRNA.

RNA is a nucleic acid that plays a central role in gene coding and gene regulation processes as well as in the process of protein synthesis [64]. The information contained in DNA is, in fact, transcribed into an RNA filament through “transcription”, which allows the generation of a messenger RNA (mRNA) for protein-coding genes, or of ncRNA (rRNA, tRNA, miRNA, siRNA, snoRNA, snRNA, etc.) for RNA-coding genes, as shown in Figure 2 [64,65,66,67,68].

Epigenetics refers to the set of reversible and interrelated transcriptional, post-transcriptional, and post-transductional alterations that modulate gene expression and activity [65], without changing the nucleotidic sequence of DNA [64]. For this reason, epigenetics plays a fundamental role in almost all aspects of development and is implicated in the onset of different diseases [66]. The modifications can be inherited, consisting of specific gene variants in transcription factors or signaling molecules, but can also be triggered by non-hereditary contributors such as dietary habits and the environment [67]. The mechanisms involved are as follows: (I) DNA methylation: DNA methylation occurs mostly in CpG islands, normally located in the promoter region of house-keeping genes and in some tissue-specific genes and consists of the addition of a methyl group to the carbon-5 of cytosine. Usually, this mechanism is associated with gene silencing. In fact, hypermethylation of CpG islands corresponds to transcription inhibition while hypomethylation correlates with transcription activation. (II) Chromatin remodeling processes (ATP-dependent chromatin remodelers, histone protein modifications, and histone variants): Processes that induce a change in chromatin conformation, making the promoter of a gene accessible to transcription factors. ATP-dependent remodelers are chromatin remodeling complexes that exploit the energy released by ATP hydrolysis to induce structural changes in chromatin. Post-translational histone modifications are based on chemical reactions (such as acetylation and phosphorylation). Finally, histone variant substitutions refer to the incorporation of non-canonical histones into the octamer that makes up the nucleosome (the fundamental unit of chromatin, composed of a histone octamer and the DNA). All three mechanisms have the goal of weakening histone–DNA interactions, inducing the displacement of the nucleosome and, therefore, the activation of gene transcription. (III) RNA regulatory mechanisms (non-coding RNAs, miRNAs, antisense RNAs, riboswitches, and others): Long non-coding RNAs (lnRNAs) can bind regulatory proteins or transcriptional factors or interact directly with DNA/RNA molecules, influencing gene expression. Similarly, miRNAs are small RNAs derived from primary miRNAs (pri-miRNAs), larger precursors that are in turn expressed by specific non-coding genes that contain one or more miRNAs from the intronic regions of protein-coding genes. Several different miRNAs can bind a target mRNA, influencing its stability and translatability.

Several epigenetic aberrant patterns are involved in the pathogenesis of congenital heart disease (CHD). Grunert M. et al. performed an analysis of the methylation profile of myocardial biopsies obtained from patients with Tetralogy of Fallot (ToF) or ventricular septal defects (VSDs) and found a hypermethylation of several promoters, including that of the SCO2 (Synthesis Of Cytochrome C Oxidase 2) gene, whose abnormal methylation could induce a delay in the terminal differentiation of cardiac cells [69]. Another study reported a correlation between the hypermethylation of the EGFR, EVC2, TBX5, and CFC1B genes and the onset of ToF [70]. Hypermethylated CpGs of the GATA4 (GATA Binding Protein 4) body gene and higher expression levels of the GATA4 transcript were also detected in the cardiac tissue of fetuses with syndromic and non-syndromic CHD, leading to the consideration of these modifications as favorable to the pathogenesis of the malformation [71]. The ZFPM2 (zinc finger protein, multitype 2 protein, a protein involved in diaphragm and cardiovascular system development) promoter region was the result in hypermethylated patients as well in patients with ToF [72]. Finally, research reported significantly higher methylation levels of the RXRA (retinoid X receptor A) gene promoter region in the right ventricular outflow tract myocardium of patients with TOF compared to a control group [4]. Aberrant DNA methylation is not the only mechanism potentially involved in CHDs. It was found that DPF3 (Double PHD Fingers 3), normally involved in histone modification by binding methylated lysine residues of H3K4 (histone H3 on lysine 4), was upregulated in the right ventricular myocardium of patients with ToF [4]. A study demonstrated the key role played by Dot1L (Dot1-like histone lysine methyltransferase) in cardiac development as the knockout model was lethal in the embryonic stage [4]. Alterations in the expression levels of the EP300 (histone acetyltransferase p300), KAT2A (lysine acetyltransferase 2A), and HAT1 (human histone acetyltransferase 1) acetylases also seem to play a role in the onset of CHDs with ASDs (atrial septal defects), VSDs, AVSDs (atrioventricular septal defects), and valve dysplasia [73,74]. Moreover, a mouse model study conducted by Lewandowski SL et al. showed severe cardiac developmental defects and embryonic lethality in embryos lacking histone deacetylase 3 (HDAC3) [75].

Several single-nucleotide polymorphisms (SNPs) seem to be related to the development of CHDs as well. It was reported that a reduction in MTHFR (methylenetetrahydrofolate reductase) enzyme activity increased plasma homocysteine levels, indirectly contributing to the onset of CHDs, as hyperhomocysteinemia was positively associated with CHDs [76,77,78]. Specifically, studies detected a correlation between MTHFR C677T gene polymorphism and CHDs [76,79]. However, further research is needed since Van Beynum et al. [80] reported no association between MTHFR C677T gene polymorphism and the risk of CHDs. The study conducted by Van Beynum et al. [81] in children with CHDs showed a positive correlation between eNOS (the endothelial nitric oxide synthase gene, encoding a protein involved in cardiac development [82]) 894G>T polymorphism and an increased risk of CHDs. The influence exerted by VEGF (vascular endothelial growth factor) polymorphisms cannot be ruled out either, although research has produced conflicting results: the study conducted by Lambrechts et al. reported that C2578A, G1154A, and C634G VEGF polymorphisms led to a lower level of VEGF, thus increasing the risk of ToF [83]. On the contrary, the meta-analysis conducted by Griffin et al. reported no association between VEGF polymorphism and CHDs [84]. Finally, researchers also evaluated alterations in copy number variations (CNVs), demonstrating the possible influence exerted by pathogenic CNVs in the etiology of both syndromic and isolated CHDs [85,86,87].

miRNAs are small RNAs of 22–24 nucleotides which are evolutionally conserved. miRNAs are of particular interest due to the important role they appear to play in gene regulation, particularly in the control they exert on mRNAs involved in important fundamentals such as cell proliferation and differentiation, apoptosis, and development, to the point of inducing researchers to investigate their etiopathogenetic functions and hypothesize their applications in the therapeutic field (as these factors are known for gene silencing methodologies via the RNA interference, RNAi) [64]. Primitive forms of mi-RNAs are transcripted into a hairpin structure which starts the maturation process into the cell nucleus, and this structure is therefore transported into the cytosol to be cut into the mature form by Dicer, an RNA-ase III endonuclease. mi-RNAs have been shown to negatively regulate gene expression by interacting with mRNA transcript 3’UTRs, thereafter promoting mRNA degradation, transcript deadenylation, translation inhibition, or sequestration in the processing body [88]. The significance of miRNAs in cardiac development was initially revealed through a study in which the deletion of two distinct exons of the Dicer gene in mouse embryos disrupted angiogenesis and resulted in embryonic lethality. Subsequent research has consistently confirmed that Dicer plays a vital role in regulating vascular signaling pathways [89]. It is evident that a dysregulation in the expression of miRNA progenitor cells of the developing heart leads to congenital or structural defects resulting from poor cell migration, proliferation, and specification into inappropriate cell types. This assumption is strongly supported by extensive evidence demonstrating the essential functions of specific miRNAs throughout the various stages of heart development, as observed in both in vitro and in vivo experimental systems. It is therefore reasonable to infer that miRNAs play an equally critical role in human cardiogenesis.

Studies have demonstrated that miRNAs regulate cardiac development by targeting various transcription factors, including TBX2, NKX2, CSPG2, and NOTCH1, particularly in zebrafish models [90]. The miR-1 (miR-1-1 and miR-1-2) and miR-133 (miR-133a-1, miR-133a-2, and miR-133b) families are key regulators during the initial stages of cardiac development. miR-1 supports the differentiation of embryonic stem cells into cardiomyocytes, whereas miR-133 counteracts this process. Additionally, both miRNAs modulate the function of the transcription factor serum response factor (SRF), further influencing cardiac gene expression [91]. SRF, involved in cell cycle progression, is regarded as one regulator of the cardiac and smooth muscle differentiation genes [92]. miR-1 represents a significant portion—around 40%—of total cardiac miRNA content and regulates key transcription factors such as IRX5 and Hand2 [93]. Similarly, miR-128a plays a role in the early phases of heart formation by promoting the differentiation of cardiomyocyte progenitor cells into distinct cardiomyocyte subtypes [94].

miR-218 plays a regulatory role by modulating TBX5 expression, whose overexpression has been shown to interfere with proper heart tube formation [95]. Additionally, members of the myomiR family, such as miR-208a and miR-499, are essential in cardiac development and are encoded within the MYH6 and MYH7 genes. miR-208a, in particular, enhances the expression of CX40, HOPX, and GATA4, all of which are key factors in shaping the cardiac gene expression landscape [96]. In addition, MiR-499 increases the differentiation of cardiac progenitors to cardiomyocytes suppressing SOX6 [97]. The miR-17-92 cluster has been shown to promote myocardial differentiation by targeting key regulators such as ISL1 and TBX1 in cardiac progenitor cells. Notably, miR-19, a component of this cluster, appears capable of inducing abnormal heart development in progenitor cells, particularly in zebrafish models [98]. Moreover, cardiac sidedness and particularly those processes regulated by the Prrx1 transcription factor are modulated by miR-34a and miR-92a [99]. Angiogenesis is controlled by multiple miRNAs. MiR-143 and miR-145 are transcribed as a bicistronic cluster and control smooth muscle cell development. While miRNA145 supports vascular smooth muscle cell differentiation, it also represses proliferation in cardiac defects. Conversely, miR-143 functions by directly suppressing Elk1, a factor involved in promoting the proliferation of vascular smooth muscle cells. The expression of miR-143, along with miR-145, can be upregulated by bone morphogenetic proteins (BMPs) and transforming growth factor beta-1 (TGF-β1) [100]. Other miRNAs guide endothelial cell formation, in particular miR-126, which is an endothelial-specific miRNA [101].

## 4. Modulation of mRNA Expression in Congenital Heart Diseases

Numerous studies have demonstrated that miRNAs are required for proper heart development and function, and that distinct miRNA expression profiles can specifically and broadly affect the cell signaling pathways associated with heart defects. A summary of all key studies on miRNA expression and congenital heart disease (CHD) is provided in Table 1.

### 4.1. Left Obstructive Heart Disease (LVOTO)

To date, only one study in the literature has focused on treating the modulation of mi-RNAs in LVOTO, with particular regard to hypoplastic left heart syndrome (HLHS).

The study by Suharov et al. [102] analyzed the expression profile of miRNAs in the right ventricle of patients with HLHS, demonstrating a unique pattern in these patients. This paper has demonstrated that numerous miRNAs are differentially regulated in patients with HLHS, many of which have not previously been associated with cardiovascular diseases. Notably, the expression of some of these miRNAs varied across different stages of the disease and surgical palliation. Specifically, the altered expression of miR-204 and miR-137-3p was observed during stages I and III of palliation. Additionally, the levels of miR-100, miR-99, and miR-145 were normalized by stage III. These findings suggest that volume overload may play a significant role in regulating miRNA expression. These miRNAs target various transcription factors (TFs): In stage I, the expression of QKI, CDK6, SOX11, BAZ2A, FOG2, and GATA6 was increased but returned to baseline levels by stage III. In contrast, the expression of GATA4 and dHAND was decreased in the same group. This supports the idea that while some miRNAs are modulated by changes in volume load on the right ventricle (RV), others appear to be independent of volume load. Importantly, the unloading of the RV in stage III of palliation leads to a reversal of these changes, suggesting that miRNA modulation is primarily a compensatory mechanism in response to increased volume load on the RV.

In conclusion, miRNA target prediction analyses have identified several genes implicated in cardiac development and pathology as potential targets of dysregulated miRNAs in hypoplastic left heart syndrome (HLHS). These include Quaking (QKI), Friends of GATA 2 (FOG-2), cyclin-dependent kinase 6 (CDK6), and SRY-box transcription factor 11 (Sox11).

### 4.2. Bicuspid Aortic Valve

Bicuspid Aortic Valve (BAV) represents a frequent congenital heart defect, affecting approximately 1–2% of the general population. It is a leading cause of aortic stenosis and regurgitation and is often associated with an elevated risk of developing thoracic aortic aneurysms [1]. Since its prevalence is very high compared to other CHDs and its consequences stretch mainly into the adulthood, miRNA modulation in BAV has been the object of intense research compared to other CHDs. Actually, by only considering patients with thoracic aneurysm and control aortic tissues, a combined list of more than 208 differentially expressed miRNAs has been observed [107]. In addition, BAV has been one of the first cardiovascular disease to be studied; already in 2010 Nigam et al. investigated the association of miRNAs and BAV. In this study, they were able to show that miR-26a, miR-195 and miR-30b levels were significantly reduced [103].

Yanagawa et al. [104] also identified distinct miRNA profiles in a small cohort of human BAV leaflets compared to controls with a tricuspid aortic valve (TAV). They identified eight miRNAs which were upregulated and 27 miRNAs which were downregulated in patients with BAV, the most significant of which were miR-141 and miR14.

Moreover, many efforts have been directed toward the modulation of miRNAs in the context of BAV and the development of aortic aneurysms, since miRNAs have the potential to be used as useful markers of disease progression and, more importantly, could be helpful for risk stratification of patients.

In 2020, a study investigating miRNAs linked to aortopathy in patients undergoing aortic valve surgery revealed an inverse relationship between circulating miRNA levels and aortic diameter in BAV-related aortopathy, with the strongest associations observed for miR-17, miR-20a, and miR-106a [108].

Borghini et al. [107] investigated the involvement of miRNAs in thoracic aortic aneurysm (TAA) among patients with Bicuspid Aortic Valve (BAV) by applying next-generation sequencing to profile the miRNome in TAA tissues from both BAV and tricuspid aortic valve (TAV) patients. This line of research appears promising, as most miRNAs identified in BAV cases have so far been reported in isolated studies, and comprehensive validation remains necessary. When compared to TAV samples, twelve known miRNAs exhibited differential expression in BAV tissues. Pathways implicated in aneurysm development included the Hippo signaling, ErbB signaling, TGF-β signaling, and focal adhesion pathways.

Sophocleous et al. [105] demonstrated that five miRNAs (miR-128-3p, miR-210-3p, miR-150-5p, miR-199b-5p, and miR-21-5p) were differentially expressed across the aortic arch circumference. miR-128-3p, miR-150-5p, and miR-199b-5p were found to have an important role in the expression of eight common target genes involved in the vascular endothelial growth factor signaling, Hippo signaling, and arachidonic acid pathways.

Alongside these findings in dilated segments of aortic tissue, there were decreased elastic fiber levels and elastic layer thickness. Furthermore, in a subset of patients, a four-dimensional cardiac magnetic resonance (CMR) scan was performed showing an increase in wall shear stress (WSS) at the anterior/right wall segments, where the differentially expressed miRNAs and decreased elastic fibers were found [105].

Another group investigated the correlation between miRNAs in the peripheral blood. Naito et al. examined patients undergoing elective aortic valve repair or replacement, with or without proximal aortic replacement, and identified a significant correlation between the peripheral blood levels and the aortic tissue expression of miR-21 and miR-145. These findings supported the hypothesis that circulating miRNAs could serve as biomarkers of remodeling processes in the proximal aorta among BAV patients [106].

Different miRNA expression in aortic dilation has also been studied exceptionally in pediatric patients with BAV. In the study published by Antequera-González and his group [109], 40% of patients had a dilated aorta and miR-130a expression was found to be significantly lower in these patients. Additionally, plasma levels of miR-130a were found to inversely correlate with ascending aorta and aortic root z scores. Enrichment analyses indicated that miR-130a targets were associated with the TGF-β signaling pathway. These findings suggest that plasma miR-130a could serve as a potential biomarker to help distinguish between low- and high-risk BAV children.

More recently, miRNA-17-5p, hsa-let-7e, and miRNA-196a-5p were found to be mostly increased in patients with BAV and aortic dilatation who underwent aortic valve replacement for aortic stenosis [110]. The downregulation of miRNA-17a-5p and the upregulation of miR-Let-7e-5p and miR-196-5p were related to an increased risk of aortic dilation risk. miRNA-196-5p had, at last, a positive correlation with valvular calcification.

### 4.3. Septation Defects

#### 4.3.1. Ventricular Septal Defects

VSDs are discontinuations in the septal wall between the left and right ventricles and these defects represent the most common congenital heart defect, accounting for approximately 20–40% of all CHDs [1]. Smaller defects may be asymptomatic and close spontaneously, whereas large defects can lead to severe heart failure due to significant left-to-right shunt resulting in pulmonary overflow, left ventricular overload, and subsequent pulmonary hypertension.

The genetic basis of a ventricular septal defect (VSD) is highly complex and remarkably heterogeneous. Associations have been identified with chromosomal abnormalities, including aneuploidies and structural variations, as well as with rare point mutations in multiple genes. VSDs are observed in well-characterized genetic syndromes, such as DiGeorge and Holt–Oram syndromes, and has been linked to mutations in genes encoding key cardiac transcription factors like NKX2-5, GATA4, and CFC1 [135].

After initial microarray screening, Li et al. in 2013 compared the expression of a set of miRNAs in the heart tissues of patients with VSDs and healthy controls, finding that in patient samples, miR-1-1 expression was decreased, whereas miR-181c expression was increased. Inversely, the expression of GJA1 and SOX9, target genes of miR-1-1, was increased, while the expression of BMPR2, a target gene of miR-181c, was decreased [111]. SOX9 and BMPR2 are involved in the formation of valves and septation of the heart [111]. Animal studies have shown that miR-1 knockout mice have an increased risk of VSDs [136,137].

Li et al. [111], in a subsequent study in 2014, also reported that circulating miRNA profiles obtained from plasma samples of VSD patients were characterized by the upregulation of hsa-miR-498 and the downregulation of hsa-let-7e-5p, hsa-miR-155-5p, hsa-miR-222-3p, hsa-miR-379-5p, hsa-miR-409-3p, hsa-miR-433, and hsa-miR- 487b [113]. miR-let-7e-5p, miR-222-3p, and miR-433 were found to target genes related to cardiac development such as NOTCH1, HAND1, ZFPM2, and GATA3.

Circulating miRNAs hold potential as prenatal biomarkers for congenital heart defects (CHDs) due to their stability in maternal blood and their ability to cross the placental barrier. Zhu et al. [112] proposed that maternal serum miRNAs could serve as non-invasive markers for the early prenatal detection of fetal CHDs. In 2013, their group analyzed miRNA profiles in serum samples from pregnant women carrying fetuses diagnosed with ASDs, VSDs, or TOF, compared to controls with normal pregnancies. They observed that elevated levels of hsa-miR-19b and hsa-miR-29c were particularly associated with VSDs [112]. The miR-29 family suppresses excess collagen expression, and it may also promote cardiac hypertrophy and CHD development; miR-29c-3p, in particular, regulates AKT3 gene expression whereas miR-29b inhibits cardiomyocyte proliferation via NOTCH2 [138].

In 2019, Gu et al. [119] identified four distinct maternal circulating miRNA profiles capable of distinguishing fetuses with VSDs, TOF, single ventricles, and persistent truncus arteriosus from healthy controls. Specifically, maternal serum samples from pregnancies affected by VSDs exhibited elevated levels of miR-1275 and miR-3664-3p, alongside the decreased expression of miR-142-5p and miR-4666a-3p. Interestingly, these miRNAs were rapidly reduced in maternal serum after delivery as compared to before delivery. miR-142 plays an important role in cardiac hypertrophy, whereas miR-1275 has been associated with coronary artery disease and myocardiocyte apoptosis [139,140]. The roles of miR-4666a-3p and miR-3664-3p in cardiac diseases are not well known yet.

In 2021, Jin et al. [120] described miRNA profiles in serum samples obtained from pregnant women who had fetuses with VSDs. The most important finding was the reduced expression of hsa-miR-146a-5p, which was found to effectively distinguish cases of fetuses with VSDs from controls. Target genes of miR-146a-5p, such as PMAIP1, NUMB, ERBB4, IRAK1, and CCL5, were related to cardiac development and morphogenesis.

#### 4.3.2. Atrial Septal Defects

Atrial septal defects (ASDs) represent the second most common form of CHDs, accounting for approximately 10–15% of all cases [1]. These anomalies permit abnormal communication between the left and right atria, typically resulting in a left-to-right shunt. Moderate to large shunts can eventually cause volume overload, heart failure, and pulmonary arterial hypertension. While most ASDs occur sporadically, some cases are linked to well-characterized genetic syndromes, such as Holt–Oram syndrome, or exhibit familial inheritance patterns, primarily being autosomal dominant. Mutations affecting genes that encode cardiac transcription factors (e.g., NKX2-5, GATA4) and structural proteins (e.g., MYH6, ACTC1) have been identified in familial ASD cases [141,142,143]. More recently, research has highlighted that alterations in gene expression at the transcriptomic level, rather than solely gene mutations, may play a critical role in ASD pathogenesis.

Zhu et al. [112] in 2013 found that in pregnant women with a fetus with ASDs, three circulating miRNAs were significantly upregulated in maternal serum compared to those with normal fetuses: hsa-miR-19b, hsa-miR-29c, and hsa-miR-375.

In 2018, Song et al. [117] identified the significant upregulation of hsa-let-7a, hsa-let- 7b, and miR-486 in children with ASDs, VSDs, and AVSDs. hsa-miR-let-7a and hsa-miR-let-7b were specifically overexpressed in ASD children. A similar expression profile of hsa-let-7a and hsa-let-7b was discovered in mothers of children with ASDs. On the other hand, the hsa-miR-486 level was significantly higher in all ASD, VSD, and AVSD groups.

In 2019, Han et al. [118] reported the significant upregulation of the miR-17-92, miR-106b-25, and miR-503/424 clusters, alongside the downregulation of the miR-29 and miR-143/145 clusters, in atrial septal tissues from sporadic ASD patients compared to healthy controls. These miRNA groups are critically involved in the key signaling pathways required for proper cardiac development and morphogenesis.

Interestingly, even single-nucleotide polymorphisms in miRNA-machinery genes may impact miRNA processing efficiency or function [144].

In 2016, Yu et al. [115] reported that a single-nucleotide polymorphism (SNP) in miR-196a2—specifically rs11614913 T>C—was linked to the occurrence of sporadic ASDs. Individuals carrying the CC or CT genotypes exhibited a lower risk of ASDs compared to those with the wild-type T allele, suggesting that the C allele may exert a protective effect against ASD development. Further studies have shown that this SNP can influence the expression of miR-196a2, enhancing its effects on cellular differentiation and morphogenesis [145].

In 2016, Wang F et al. [114] discovered a novel mutation—c.*1784T>C—located in the 3′UTR of the ACTC1 gene among patients with familial isolated secundum ASDs. This mutation entailed a new miR-139-5p target site with enhanced binding affinity, resulting in the decreased expression of the ACTC1 gene.

In 2017, Wang Y et al. [116] also found that a genetic polymorphism—rs6489956 C>T—at the 3′UTR region of the TBX gene was responsible for increased susceptibility to ASDs, as the CT and TT genotypes were associated with an elevated risk of ASDs compared to the wild CC genotype. Compared to the C allele, the T allele showed increased binding affinity to miR-9 and miR-30a, leading to the downregulation of TBX5 expression at the transcriptional and translational levels, respectively.

In 2022, Jia et al. [121] identified a genetic mutation—c.335-1G>A—affecting the splicing region of the NKX2-5 gene in patients with familial ASDs. This mutation appeared to suppress the expression of miR-19a/b, resulting in the upregulation of PYK2, a cytoskeletal protein and tyrosine kinase involved in key cellular processes such as cardiomyocyte proliferation, differentiation, and apoptosis.

#### 4.3.3. Atrioventricular Septal Defects

As mentioned previously, in 2018, Song et al. identified the significant upregulation of circulating hsa-miR-486 in serum samples from children with ASDs, VSDs, and AVSDs [143].

Almost half of all AVSDs occur in patients with Down Syndrome and, conversely, approximately 25% of these patients have AVSDs [146]. It has been found that Down Syndrome was linked to five miRNAs, including miR-99a, miR-let-7c, miR-125b-2, miR-155, and miR-802, all of which were identified on human chromosome 21 and were overexpressed in the cardiac tissue of patients with trisomy 21 [147,148]. Subsequent studies have reported that the overexpression of the miR-99a/let-7c cluster may lead to the reduced expression of their biological targets in the fetal heart tissue from individuals with Down Syndrome, suggesting a potential pathogenic role of these miRNAs in the development of congenital heart defects in this population [149,150].

### 4.4. Tetralogy of Fallot and Other Conotruncal Anomalies

Conotruncal malformations are a group of CHDs that affect the heart’s outflow tract and great vessels and share similar intracardiac pathology. These anomalies include Tetralogy of Fallot (TOF), pulmonary atresia (PA) with VSDs, double outlet right ventricle (DORV), and the transposition of the great arterial vessels (TGA).

#### Tetralogy of Fallot

Tetralogy of Fallot is the most common form of cyanotic CHD and represents 5–8% of all CHDs (1). This anomaly is characterized by the tetrad of (1) conoventricular VSDs, (2) overriding aorta, (3) right ventricular outflow obstruction, and (4) right ventricular hypertrophy. Without surgery, patients have poor prognosis with 33% mortality within the first year of life and a survival rate of 11% at 20 years, 6% at 30 years, and 3% at 40 years [151]. Although non-syndromic TOF is relatively common and clinically significant, its underlying etiology remains poorly understood. It is likely driven by a complex interplay of subtle genetic, structural genomic, and epigenetic changes, together with environmental factors. In recent years, miRNAs have emerged as potential contributors to TOF pathogenesis, with several studies reporting dysregulated miRNA profiles in affected patients.

In 2012, O’Brien et al. [123] analyzed microRNA expression in right ventricular myocardial tissue from infants with non-syndromic TOF and compared it to tissue from infants with structurally normal hearts. They identified 61 miRNAs with significantly altered expression between the groups. Among these, miR-1275, miR-27b, miR-421, miR-1201, and miR-122 were highlighted as potential regulators of the genes essential for cardiac development, suggesting a possible role in the pathogenesis of TOF. In 2014, in a subsequent study, Bittel and Colleagues [127] performed a follow-up study focusing on miR-421 by knocking-down or overexpressing this miRNA in primary cells derived from the right ventricular myocardium of infants with TOF and healthy controls, respectively. Their analysis revealed a significant inverse relationship between miR-421 expression and SOX4 levels—a critical transcription factor involved in the NOTCH signaling pathway, which plays a key role in the development of the cardiac outflow tract.

The altered expression of connexin-43 has been implicated in the development of conotruncal anomalies [152]. Wu and colleagues investigated ten candidate miRNAs potentially regulating connexin-43 and found that miR-1 and miR-206 were significantly downregulated in patients with TOF compared to healthy controls, suggesting their involvement in the disease’s molecular pathogenesis [124].

In 2013, Zhang et al. [125] showed 18 miRNAs with significantly altered expression, and found that 16 of these targeted several genes involved in heart development: miR-146b-5p, miR-155, miR-19a, miR-222, miR-424, miR-337-5p, miR-363, miR-130b, miR-154, miR-708, miR-181c, miR-424*, miR-181d, miR-192, miR-660, miR-29c, miR-720, and miR-181a*. Furthermore, the Authors demonstrated that miR-424 targeted the NF1 and HAS2 genes, whose expression was decreased in RVOT myocardial tissues from patients with TOF, suggesting a potential pathogenetic role of this miRNA.

Zhu et al. [112] in 2013 reported that four maternal circulating miRNAs (miR-19b, miR-22, miR-29c, and miR-375) were able to detect fetal CHDs. In this study, miR-22 appeared to be specifically upregulated in TOF patients.

In 2013, He et al. [128] investigated the chronic hypoxia commonly observed in patients with cyanotic CHDs and reported that miR-138 expression was upregulated in myocardial samples from patients with cyanotic CHDs such as TOF compared to patients with acyanotic CHDs. The Authors proposed that the upregulation of miR-138 attenuated hypoxia-induced apoptosis in cardiomyocytes by directly inhibiting MLK3, a pro-apoptotic kinase, as a compensatory mechanism for cardioprotection against chronic hypoxia.

According to a 2014 study by Liang and colleagues [126], miR-940 was the most significantly downregulated miRNA in myocardial tissue from TOF patients, and was among the 75 differentially expressed miRNAs identified in comparison to healthy controls. Moreover, miR-940 showed the highest expression in the right ventricular outflow tract of normal human hearts compared to other cardiac chambers, pointing to a possible pathogenic role in TOF.

In 2018, Wang et al. [130] explored the sexual differences in small RNA (sRNA) and miRNA expression profiles in TOF patients. The Authors discovered a significant sexual difference in sRNA expression in TOF patients and found that miR-1/miR-133 accounted for the most variance in sRNA expression between the sexes.

In 2019, Grunert et al. [131] reported 172 miRNAs that were significantly differentially expressed in TOF patients. Among these, the Authors highlighted the potential pathogenetic role of five miRNAs—miR-1, miR-133b, miR-139-5p, miR-140-5p, and miR-146b-5p— targeting 18 genes essential to cardiac development and function—including KCNJ2, FBN2, SLC38A3, and TNNI1. Approximately 25% of the differentially expressed miRNAs overlapped with the results from previous studies, with only one miRNA being altered in all these studies—namely miR-222-3p, a member of the miR-221/222 cluster, which seemed to play a crucial role in heart disease by targeting genes related to TGF-β signaling [131].

In 2020, You et al. [132] analyzed microRNA expression profiles from Tetralogy of Fallot (TOF) patients using datasets from the NCBI Gene Expression Omnibus (GEO). Their study identified seven miRNAs (miR-499, miR-23b, miR-222, miR-1275, miR-93, miR-155, and miR-187) that were significantly upregulated in TOF patients compared to healthy controls. These miRNAs are known to influence cardiac development and function, and their altered expression is thought to contribute to abnormal cardiac cell proliferation, differentiation, and apoptosis in TOF pathogenesis.

In 2021, Chouvarine et al. [133] examined miRNA expression profiles in RV tissues from infants with TOF or pulmonary stenosis (PS) compared to samples from infants with ventricular septal defects but no PS to investigate right ventricular hypertrophy (RVH). The Authors identified four miRNAs (miR-31, miR-216a, miR-372, and miR-5008) as potential epigenetic regulators of right ventricular hypertrophy (RVH) in TOF with pulmonary stenosis (TOF/PS). These miRNAs were found to target genes involved in key biological processes, including glucose and lipid metabolism (SIK1, FABP4), cell surface interactions (THBS2, FN1), apoptosis (PIK3IP1, SIK1), and extracellular matrix remodeling (CTGF, IGF1). Notably, miR-31 expression was higher in male patients, whereas miR-372 was more abundant in females.

In a recent study, Yang et al. [134] analyzed the miRNA expression profiles of exosomal vesicles derived from the amniotic fluid of fetuses with TOF. The Authors identified 257 significantly dysregulated miRNAs, 25 of which targeted genes involved in Tetralogy of Fallot and congenital heart diseases. Also, the significant upregulation of miR-10a-5p was found to directly inhibit the expression of the TBX5 gene, a member of the T-box transcription factor family crucial for cardiomyocyte differentiation and heart development, potentially contributing to the pathogenesis of TOF.

Interestingly, the miRNA expression profile appears to differ in TOF infants and post-repair TOF patients. Circulating miRNAs after surgical repair are often associated with residual lesions and altered right ventricular loading conditions and could serve as potential biomarkers for disease progression in TOF patients.

In 2017, Abu-Halima et al. [129] investigated miRNA expression among long-term post-repair TOF patients, focusing on the association of miRNAs with symptomatic heart failure. The Authors reported that circulating miR-421, miR-1233-3p, and miR-625-5p were significantly lower in patients with symptomatic heart failure compared to asymptomatic individuals.

Unfortunately, there are few publications investigating miRNA expression profiles in other conotruncal malformations, which are rarer than the relatively frequent Tetralogy of Fallot, and further studies are needed to confirm the role of specific miRNAs in the development and progression of such defects.

Conotruncal anomalies frequently occur in individuals with 22q11.2 deletion syndromes, including DiGeorge and velo-cardio-facial syndromes. DiGeorge syndrome, the most common microdeletion disorder, involves the deletion of the 22q11.2 region, which encompasses the TBX1 gene, essential for normal heart development, and DGCR8, a key component of the RNA-induced silencing complex (RISC) required for miRNA biogenesis. This genetic configuration suggests that altered miRNA expression may play a role in the pathogenesis of the syndrome, potentially contributing to gene dosage sensitivity and congenital heart malformations [153].

**Figure 3:**
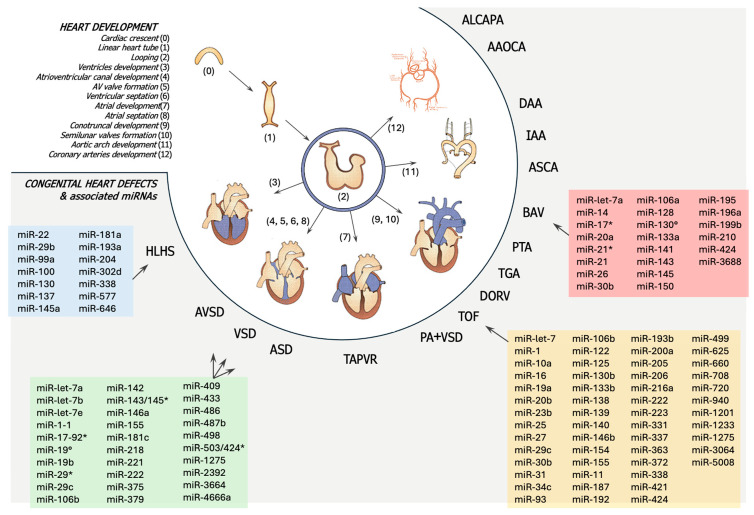
Schematic representation of heart development and distinct miRNA associated to common cardiac heart diseases (CHDs). The white section describes the stages of heart development. The grey section, with blue, green, red, and yellow boxes, outlines the miRNAs involved in the development of the corresponding congenital heart defects. AAOCA = Anomalous Aortic Origin of a Coronary Artery; ALCAPA = Anomalous Left Coronary Artery from the Pulmonary Artery; ASCA = Aberrant Subclavian Artery; ASD = Atrial Septal Defect; AVSD = Atrioventricular Septal Defect; BAV = Bicuspid Aortic Valve; DAA = Double Aortic Arch; DORV = Double Outlet Right Ventricle; HLHA = Hypoplastic Left Heart Syndrome; IAA = Interrupted Aortic Arch; PA = Pulmonary Atresia; PTA = Persistent Truncus Arteriosus; TAPVR = Total Anomalous Pulmonary Venous Return; TGA = Transposition of the Great Arteries; TOF = Tetralogy of Fallot; VSD = Ventricular Septal Defect. The asterisk (*) indicates the passenger strand (miRNA) of the microRNA duplex.

In Figure 3, miRNA expression in CHDs is schematized.

## 5. Future Perspectives on miRNAs as Biomarkers

MiRNAs are also emerging as plausible sensitive biomarkers in different cardiac pathological conditions.

In this context, the identification of placenta-derived miRNAs in maternal plasma has been linked to congenital heart defects (CHDs), paving the way for promising advancements in early, non-invasive prenatal screening and potentially in developing preventive strategies.

Zhu et al. employed sequencing by oligonucleotide ligation and detection (SOLiD) technology to systematically analyze maternal serum miRNAs, hypothesizing their potential as early biomarkers for the prenatal detection of fetal CHDs [112]. Their study included 60 participants—30 pregnant women with CHD-affected fetuses and 30 controls with normal pregnancies. They identified four miRNAs—miR-19b, miR-22, miR-29c, and miR-375—that were significantly upregulated in the maternal serum of CHD cases. Individually, these miRNAs demonstrated sensitivity ranging from 55.6% to 77.8% and specificity between 66.7% and 88.9%. Notably, combining all four biomarkers enhanced diagnostic accuracy, suggesting a promising multi-marker strategy for CHD screening.

A more recent investigation by Gu et al. [119] involved 110 pregnant women—50 carrying fetuses with CHDs and 60 being healthy controls. Through microarray analysis, they identified 38 serum miRNAs that were differentially expressed between the two groups. Among these, a diagnostic panel of four miRNAs—miR-142-5p, miR-1275, miR-4666a-3p, and miR-3664-3p—was proposed as being capable of distinguishing CHD cases from controls. Interestingly, ten of the dysregulated miRNAs exhibited significantly lower expression levels in maternal serum after delivery compared to the levels measured during pregnancy.

It is clear that measuring miRNAs in a sample blood or urine test is a minimally invasive investigation. Identifying disease-specific and stable miRNAs may provide a fundamental tool to detect CHDs both antenatally and postnatally. Further research is required to accurately explore the possibility that miRNAs can be used in clinical practice for the prenatal detection of CHDs.

## 6. Limitations

Several limitations should be considered when interpreting the findings of this review. As a narrative rather than a systematic review, the analysis presents a qualitative synthesis of the selected literature, without the rigor of standardized protocols. This approach can introduce selection bias and may limit the comprehensiveness of the review. For example, our search of the literature was restricted to studies indexed in PubMed and Scopus, potentially excluding relevant publications available through other databases or platforms.

Additionally, the literature reports had some issues that must still be overcome, mostly related to sample size of these studies, the huge heterogeneity of CHDs, and the possible variability within the populations themselves.

Moreover, longitudinal data during pregnancy are still limited. To advance future research, we advocate for the implementation of well-designed studies, including both maternal and fetal analyses, to clarify the role of mRNA expression regulation in CHDs and to explore its potential as a promising biomarker.

Finally, although the literature on miRNA expression in syndromic CHDs remains limited and mostly focused on Down Syndrome, studying genetic syndromes may offer valuable insights into the molecular mechanisms underlying CHDs. DiGeorge syndrome, for instance, results from the deletion of the critical region 22q11.2 on chromosome 22, which encodes a component of the RNA-induced silencing complex essential for miRNA biogenesis [122]. This deletion leads to the haploinsufficiency of the complex, and many individuals with DiGeorge syndrome present with CHDs. This association suggests that multiple miRNAs are involved and that miRNA dysregulation may contribute to gene dosage sensitivity in this condition [154].

Furthermore, embryological connections between cardiac and craniofacial anomalies exist both molecularly and clinically [155]. For example, the deletion of Dicer in neural crest cells—key contributors to both cardiac and facial development—can result in severe cardio-craniofacial defects. Several syndromes, including Noonan syndrome, DiGeorge syndrome, LEOPARD syndrome, cardio-facio-cutaneous syndrome, and Costello syndrome, display the overlapping features of cardiac and craniofacial malformations [156,157]. Investigating these syndromes and their associated miRNA expression patterns could thus provide a deeper understanding of the shared developmental pathways and molecular disruptions leading to CHDs [158].

## 7. Conclusions

Understanding the molecular mechanisms underlying congenital heart disease represents a crucial challenge for improving early diagnosis and the development of targeted therapies. In this context, the regulation of mRNA expression emerges as a key factor in the pathogenesis of these conditions, paving the way for the identification of novel molecular biomarkers. Alterations in transcriptional profiles could, in fact, offer innovative and highly specific tools for risk stratification and the clinical monitoring of patients. Although further studies are needed to validate the efficacy and clinical applicability of these biomarkers, the mRNA-based approach stands out as a promising perspective for precision medicine in the context of CHDs.

## Figures and Tables

**Table 1:** Key studies on miRNA expression related to congenital heart diseases.

Author	Type of Study	Population	Tissue Type	miRNA	Main Results
**Left Obstructive Heart Disease (LVOTO)**					
Sucharov et al., 2015 [102]	Case–control study	Patients with diagnosis of HLHS < 13 years who underwent cardiac transplantation	Right ventricle myocardial cells	Upregulated: miR-22, mi-R-181a, mi-R29b, mi-R130, mi-R302d, and m-R646; downregulated:mi-R577, mi-R193a-5p, andmi-R338-5P; mi-R differently regulated according to stage: niR-145a, mi-R100, mi-R99a, mi-R-204, and mi-R137-3p	Significant correlation between right ventricle and miRs.
**Bicuspid Aortic Valve (BAV)**					
Nigam et al., 2010 [103]	Case–control study	Adult patients with BAV undergoing aortic valve replacement	Valval leaflet	Downregulated: miR 26, mir 195, and miR30B; Upregulated: miR14.5	Significant correlation between peripheral miRs and aortic replacement.
Yanagawa et al., 2012 [104]	Case–control study	Adult patients with BAV undergoing aortic valve replacement	Valvar leaflet	Downregulated: miR-141 and miR14	Significant correlation between peripheral miRs and aortic replacement.
Sophocleus et al., 2022 [105]	Prospective study	Patients with BAV and aortopathy	Tissue biopsy	miR-128-3p, miR-210-3p, miR-150-5p, miR-199b-5p, and miR-21-5p	Correlation between miRNAs and aortic dilation.
Naito et al., 2022 [106]	Prospective study	Patients who received elective aortic valve repair/replacement ± proximal aortic replacement to BAV disease	Blood serum and aortic tissue	miR-21 miR-133a miR-143, and miR-145	Significant correlation between peripheral whole blood and aortic tissue miRs.
Borghini et al., 2017 [107]	Prospective study	Patients with ascending TAA associated with BAV or TAV	Tissue specimens	Downregulation of miR-424-3p and miR-3688-3p in BAV patients compared to TAV patients	Correlation between miRs and aortic dilation.
Girdauskas et al., 2020 [108]	Prospective study	Patients who underwent aortic valve replacement	Blood serum	miR-17, miR-20a, and miR-106a	Correlation between miRs and aortic dilation.
Antequera-González et al., 2024 [109]	Prospective study	Patients with BAV < 17 years	Blood serum	miR-130a expression in plasma inversely correlated with ascending aorta and aortic root z scores	Significant correlation between miRs and ascending aorta and aortic root z scores.
Sanchez-Garcia et al., 2025 [110]	Prospective Study	Patients who underwent aortic valve replacement for aortic stenosis	Aortic tissue	miR-17-5p, hsa-let-7e, and mi-196a-5p	Significant correlation between aortic tissue miRs and aortic dilation and calcification.
**Septation defects**					
Li et al., 2013 [111]	Case–control study	Patients with CHDs who underwent repair of VSDs and 28 healthy controls	Cardiac tissue	miR-1-1 and miR-181c	Downregulation of miR-1-1 and upregulation of miR-181c in VSD patients.
Zhu et al., 2013 [112]	Case–control study	Pregnant women bearing a fetus with ASDs, VSDs, or TOF and 30 normal pregnancy cases	Maternal serum	miR-19b, miR-29c, and miR-375	Upregulation of miR-19b, miR-22, miR-29c, and miR-375 in pregnant women with fetal CHDs; miR-19b and miR-29c were found to bear correlation with VSDs, whereas miR-19b, miR-29c, and miR-375 correlated with ASDs and all four miRs with TOF.
Li et al., 2014 [113]	Case–control study	Patients with VSDs and 15 healty controls	Serum	miR-let-7e, miR-155-5p, miR-222-3p, miR-379-5p, miR-409-3p, miR-433, miR-487b, and miR-498	Circulating miR profile for patients with VSDs showed that miR-let-7e, miR-155-5p, miR-222-3p, miR-379-5p, miR-409-3p, miR-433, and miR-487b were downregulated and miR-498 was upregulated when matched to VSD-free controls.
Wang et al., 2016 [114]	Family study	Chinese family with autosomal-dominant isolated ASD; four of the five individuals in the family had a similar clinical expression and a diagnosis of ASD	Serum	miR-139-5p	c.*1784 (T>C) mutation in the 3′UTR of the ACTC1 gene in familial ASD patients entailed a new miR-139-5p target site, and miR-139-5p binding to this target site decreased ACTC1 expression.
Yu et al., 2016 [115]	Cross-sectional study	Chinese individuals	Serum	miR-196-a2	c.*1784 (T>C) mutation in the 3′UTR of the ACTC1 gene in familial ASD patients entailed a new miR-139-5p target site, and miR-139-5p binding to this target site decreased ACTC1 expression.
Wang et al., 2017 [116]	Case–control study	CHD patients and healthy controls	Serum	miR-9 * and miR-30a	rs6489956 (C>T) single-nucleotide polymorphism in the 3′UTR of the TBX5 gene was associated with ASD and VSD occurrence, as the T allele showed a higher affinity for binding to miR-9 and miR-30a compared to the C allele, thus decreasing TBX5 expression.
Song et al., 2018 [117]	Case–control study	Families, each having a child with CHDs and parents without any cardiovascular disorder, and families unaffected by cardiovascular disease as controls	Serum, maternal serum	miR-486	Upregulation of hsa-miR-let-7a, hsa-miR-let-7b, and miR-486 in children with ASDs, VSDs, and AVSDs; hsa-miR-let-7a and hsa-miR-let-7b were specifically overexpressed in ASD children and a similar expression profile was confirmed in mothers of children with ASDs. hsa-miR-486 level was significantly higher in all ASD, VSD, and AVSD groups.
Han et al., 2019 [118]	Case–control study	Infants with ASDs and normal fetuses obtained from pregnant women who underwent voluntary abortion as controls	Atrial septum	miR-29 *, miR-143/145 *, miR-17-92 *, miR-106b-25, and miR-503/424 *	Upregulation of miR-17-92, miR-106b-25, and miR-503/424 clusters and downregulation of miR-29 and miR-143/145 clusters in atrial septum tissues of sporadic ASD patients compared with healthy controls.
Gu et al., 2019 [119]	Case–control study	Pregnant women with CHD fetuses and women carrying normal fetuses	Maternal serum	miR-142-5p, miR-1275, miR-3664-3p, and miR-4666a-3p	Maternal serum of fetuses with VSDs had a higher expression of miR-1275 and miR-3664-3p and a reduced expression of miR-142-5p and miR-4666a-3p. Interestingly, these microRNAs were rapidly reduced in maternal serum after delivery as compared to before delivery.
Jin et al., 2021 [120]	Case–control study	Pregnant women bearing a fetus with VSDs and women carrying normal fetuses	Maternal serum	miR-146a	Reduced expression of hsa-miR-146a-5p was found to effectively distinguish cases of fetuses with VSDs from controls.
Jia et al., 2022 [121]	Family study	Family members with familial VSD and healthy family members	Serum	miR-146a	c.335-1 (G>A) mutation located at the splicing site of NKX2 in individuals with familial ASDs appeared to inhibit the expression of miR-19a/b which in turns upregulated PYK2, a key cytoskeletal protein and tyrosine kinase in the regulation of cell processes like cardiomyocyte proliferation, differentiation, and apoptosis.
Ramachandran et al., 2022 [122]	Cross-sectional study	CHD patients	Cardiac tissue	miR-218-5p, miR-221-3p, and miR-2392	Upregulation of miR-218-5p was associated with VSDs, whereas downregulation of miR-221-3p and miR-2392 was associated with ASDs.
Tetralogy of Fallot and conotruncal anomalies					
O’Brien et al., 2012 [123]	Case–control study	A total of 16 infants with non-syndromic TOF and 8 healthy controls	RV outflow tract myocardium	miR-1275, miR-27b, miR-421, miR-1201, miR-122, and another +56 miRNAs	A total of 61 miRNAs were found to be significantly changed in expression in the RV myocardium of children with TOF compared to normally developing controls. Of these, miR-1275, miR-27b, miR-421, miR-1201, and miR-122 were shown to potentially target genes critical to cardiac development.
Wu et al., 2014 [124]	Case–control study	Myocardial samples from 30 TOF patients and 10 healthy controls; blood samples from 200 TOF patients and 200 controls	RV myocardium	miR-1 and miR-206	Among 10 putative connexin-43-related miRNAs, miR-1 and miR-206 expression was significantly decreased in the TOF patients as compared to controls, suggesting a role of these miRNAs in the pathogenesis of the disease.
Zhang et al., 2013 [125]	Case–control study	A total of five infants with non-syndromic TOF and three healty controls	RV outflow tract myocardium	miR-146b-5p, miR-155, miR-19a, miR-222, miR-424, miR-337-5p, miR-363, miR-130b, miR-154, miR-708, miR-181c, miR-424 *, miR-181d, miR-192, miR-660, miR-29c, miR-720, and miR-181a *	A total of 18 miRNAs had significantly altered expression, and it was found that 16 of these targeted several genes involved in heart development. miR-424 targeted the NF1 and HAS2 genes, whose expression was decreased in RVOT myocardial tissues from patients with TOF, suggesting a pathogenetic role.
Zhu et al., 2013 [112]	Case–control study	A total of 30 pregnant women bearing a fetus with ASDs, VSDs, or TOF and 30 normal pregnancy controls	Maternal serum	miR-19b, miR-22, miR-29c, and miR-375	Upregulation of miR-19b, miR-22, miR-29c, and miR-375 in pregnant women with fetal CHDs; miR-22 appeared to be specifically upregulated in TOF patients.
He at al., 2013 [126]	Experimental study	A total of 21 CHD patients, 10 with cyanotic CHDs (9 TOF, 1 PA + VSD) and 11 with acyanotic CHDs (VSD + RVOTO)	Right ventricular myocardium	miR-138	Hypoxia induced upregulation of miR-138, which decreased the protein level of its target MLK3 and attenuated hypoxia-induced apoptosis in cardiomyocytes.
Bitttel et al., 2014 [127]	Experimental study	Primary cells from right ventricular tissue of 16 infants with TOF and of 8 healthy controls	Right ventricular myocardium	miR-421	miR-421 modulated the expression of genes of importance to heart development such as SOX4 and could play a role in the pathogenesis of cardiac defects.
Liang et al., 2014 [128]	Case–control study	A total of 26 TOF patients and 15 healthy individuals	Right ventricular myocardium	miR-940 and another + 74 miRNAs	A total of 75 miRNAs were found to be differentially expressed between TOF patients and healthy controls. miR-940 was the most downregulated miRNA in the myocardium from patients with TOF and was the only one to be most highly expressed in the normal human RVOT compared to other chambers within the heart, suggesting a potential pathogenetic role.
Abu-Halima et al., 2017 [129]	Case–control study	A total of 37 long-term post-repair TOF patients and 15 healthy controls	Serum	miR-421, miR-1233-3p, and miR-625-5p	Expression levels of miR-421, miR-1233-3p, and miR-625-5p were lower in TOF patients with symptomatic right heart failure, potentially indicating disease progression in these patients.
Wang et al., 2018 [130]	Observational study	A total of five female TOF patients and five male TOF patients	Right ventricular myocardium	miR-1/miR-133 *	Significant sexual differences in small RNA expression in TOF patients; miR-1/miR-133 cluster accounted for the greatest variance in sRNA expression between the sexes.
Grunert et al., 2019 [131]	Case–control study	A total of 22 isolated TOF patients and 3 healthy controls	Right ventricular myocardium	miR-1, miR-133b, miR-139-5p, miR-140-5p, miR-146b-5p, and another +167 miRNAs	A total of 172 miRNAs were significantly differentially expressed in TOF patients. Among these, the Authors highlighted the potential pathogenetic role of five miRNAs—miR-1, miR-133b, miR-139-5p, miR-140-5p, and miR-146b-5p—targeting 18 genes essential to cardiac development and function—including KCNJ2, FBN2, SLC38A3, and TNNI1.
You et al., 2020 [132]	Cross-sectional study	NCBI Gene Expression Omnibus (GEO) database, consisting at the time of the study of 75 TOF patients and 32 matched healthy controls	miRNA expression profile datasets	miR-499, miR-23b, miR-222, miR-1275, miR-93, miR-155, and miR-187	Seven miRNAs were significantly upregulated in TOF patients compared to healthy controls—miR-499, miR-23b, miR-222, miR-1275, miR-93, miR-155, and miR-187. These miRNAs have been demonstrated to participate in cardiac development and function and their dysregulation is supposed to contribute to abnormal cardiac cell division, differentiation, and apoptosis in TOF pathogenesis.
Chouvarine et al., 2021 [133]	Case–control study	A total of 19 infants with TOF of PS and 8 controls affected by VSDs without PS	Right ventricular myocardium	miR-31, miR-216a, miR-372, and miR-5008	Four miRNAs (miR-31, miR-216a, miR-372, and miR-5008) were shown to be potentially involved in the epigenetic regulation of RVH in TOF/PS. Interestingly, miR-31 was more prominent in male patients, while miR-372 was predominant in females.
Yang et al., 2025 [134]	Case–control study	Five pregnant women carrying fetuses diagnosed with TOF through fetal echocardiography and five pregnant women carrying healthy fetuses	Amniotic fluid	miR-3064-5p, miR-206, miR-193b-3p, miR-205-5p, miR-10a-5p, miR-338-3p, miR-106b-5p, miR-25-3p, miR-223-3p, miR-20b-5p, miR-let-7e-5p, miR-34c-5p, miR-125b-5p, miR-200a-3p, miR-125a-5p, miR-let-7c-5p, miR-30b-5p, miR-16-5p, miR-331-3p, miR-27b-3p, miR-27a-3p, miR-let-7f-5p, miR-let-7a-5p, miR-let-7g-5p, and miR-449a	There were a total of 257 significantly dysregulated miRNAs, 25 of which targeted genes involved in Tetralogy of Fallot and congenital heart diseases. The upregulation of miR-10a-5p was found to directly inhibit the expression of the TBX5 gene, which is crucial for cardiomyocyte differentiation and heart development.

ASD, atrial septal defect; AVSD, atrioventricular septal defect; BAV, Bicuspid Aortic Valve; CHD, congenital heart defect; miR, microRNA; TOF, Tetralogy of Fallot; TAA, thoracic aortic aneurysm; TAV, tricuspid aortic valve; VSD, ventricular septal defect. The asterisk (*) indicates the passenger strand (miRNA) of the microRNA duplex.

## Data Availability

Not applicable.
